# Supplementary material for: Cloning and Functional Characterization of Dihydroflavonol 4-Reductase Gene Involved in Anthocyanidin Biosynthesis of Grape Hyacinth
Source: Int J Mol Sci. 2019 Sep 24;20(19):4743. doi: 10.3390/ijms20194743 (PMC6801978; doi:10.3390/ijms20194743)
Supplement: Supplementary file 1 [file ijms-20-04743-s001.zip › supplementary/Supplementary legends.docx]

**Supplementary Material：**

Supplementary Table S1 List of primers used in this study

Supplementary Figure S1 Cloning MaDFR gene and bioinformatics analysis. (A). Grape hyacinth (Muscari aucheri ‘Dark Eyes’). (B). The amplification of MaDFR gene from M. aucheri ‘Dark Eyes’. Lane 1 indicates amplification of the full-length of MaDFR. M, Trans 2K plus DNA marker. (C). The full length of cDNA and deduced amino acid sequence of MaDFR from M. aucheri. The amino acid sequences marked by red and black underline indicated the conserved NADPH-binding domain and substrate-binding domain, respectively.

Supplementary Figure S2 SDS-PAGE analysis and enzyme activity assay of the Site-directed mutagenesis of His-tagged MaDFR protein. (A, B) Coomassie brilliant blue stained polyacrylamide gel of MaDFRa and MaDFRb. M, protein marker. Lane 1, total protein extract of E. coli BL21 (DE3) harboring the empty pET-28a expression vector. Lane 2, total protein extract of E. coli BL21 (DE3) containing the expression plasmid pET-28a-MaDFRa/b before induction. Lane 3, total protein extract of E. coli BL21 (DE3) containing the expression plasmid pET-28a-MaDFRa/b after induction. Lane 4, the bacterium containing crude proteins extract of E. coli BL21 (DE3) containing the expression plasmid pET-28a-MaDFRa/b after induction. Lane 5, the supernatant containing crude protein extract of E. coli BL21 (DE3) containing the expression plasmid pET-28a-MaDFRa/b after induction. (C). Coomassie brilliant blue stained polyacrylamide gel of MaDFRc. M, protein marker. Lane 1, total protein extract of E. coli BL21 (DE3) harboring the empty pET-28a expression vector. Lane 2, total protein extract of E. coli BL21 (DE3) containing the expression plasmid pET-28a-MaDFRc before induction. Lane 3, total protein extract of E. coli BL21 (DE3) containing the expression plasmid pET-28a-MaDFRc after induction. Lane 4 and 5, the supernatant containing crude protein extract of E. coli BL21 (DE3) containing the expression plasmid pET-28a-MaDFRc after induction. Lane 6, the bacterium containing crude proteins extract of E. coli BL21 (DE3) containing the expression plasmid pET-28a-MaDFRafter induction. (D) Western blot analysis of the Site-directed mutagenesis of His-tagged DFR protein. M, Protein marker. Lane 1, negative control (the protein of empty pET-28a). Lane 2, MaDFRa protein. Lane 3, MaDFRb protein. Lane 4, MaDFRc protein.

Supplementary Figure S3 Schematic diagram of T-DNA region of Pcambia2300-35SMaDFR vectors. GFP, GFP fluorescence; Nos T, terminator of the nopaline synthase gene; LB and RB represented the left border and right border of the T-DNA region, respectively.
