# Supplementary material for: Cloning and Functional Characterization of Dihydroflavonol 4-Reductase Gene Involved in Anthocyanidin Biosynthesis of Grape Hyacinth
Source: Int J Mol Sci. 2019 Sep 24;20(19):4743. doi: 10.3390/ijms20194743 (PMC6801978; doi:10.3390/ijms20194743)
Supplement: Supplementary file 1 [file ijms-20-04743-s001.zip › supplementary/Supplementary Table S1.docx]

Supplementary Table S1 List of primers used in this study

| **Primer name** | **Primer sequence (5'→3')** |
| --- | --- |
| *MaDFR-F* | ATGACGATGAAGATGGAGAAGGG |
| *MaDFR-R* | CTAGTGTGAAGCAATGGGAACATC |
| *MaDFR-qRT-F* | AGCCGACTGTTGATGGAATG |
| *MaDFR-qRT-R* | ACTCGATATCACTCCATGCGCT |
| *MaActin-qRT-F* | AACATTCAGAAAGAGTCCACCC |
| *MaActin-qRT-R* | GCTTACCAGCAAAGATCAACCG |
| *NtCHS-qRT-F* | TGACACCCACTTGGATAGTTTAG |
| *NtCHS-qRT-R* | CGACCTCTGGAATTGGATCAG |
| *NtCHI-qRT-F* | CTTTTCTCGCCGCTAAATG |
| *NtCHI-qRT-R* | TTTCTGCCACCTTCTCTG |
| *NtF3H-qRT-F* | CAAGGCATGTGTGGATATGG |
| *NtF3H-qRT-R* | TGTGTCGTTTCAGTCCAAGG |
| *NtF3'H-qRT-F* | AGGCTCAACACTTCTCGT |
| *NtF3'H-qRT-R* | CATCAACTTTGGGCTTCT |
| *NtF3'5'H-qRT-F* | CGCACTACCATACTTAGGAGCCAT |
| *NtF3'5'H-qRT-R* | CAGCATCAGGAGTAGAAGCAACAG |
| *NtDFR-qRT-F* | AACCAACAGTCAGGGGAATG |
| *NtDFR-qRT-R* | TTGGACATCGACAGTTCCAG |
| *NtANS-qRT-F* | TGGCGTTGAAGCTCATACTG |
| *NtANS-qRT-R* | GGAATTAGGCACACACTTTGC |
| *NtUFGT-qRT-F* | GAGTGCATTGGATGCCTTTT |
| *NtUFGT-qRT-R* | CCAGCTCCATTAGGTCCTTG |
| *NtTubA1-qRT-F* | CTCCTATGCTCCTGTCATTTC |
| *NtTubA1-qRT-R* | GGCGAGGATCACACTTAAC |
| *NtAN2-qRT-F* | GAAGAAAGGTGCATGGACTG |
| *NtAN2-qRT-R* | TCTGCAGCTCTTTCTGCATC |
| *NtAN1a- qRT-F* | ACCATTCTCGAACACCGAAG |
| *NtAN1a- qRT-R* | TGCTAGGGCACAATGTGAAG |
| *NtAN1b- qRT-F* | CTTGAACACTTCTCAAACCGA |
| *NtAN1b- qRT-R* | TGCTAGGGCACAATGTGAAG |
| *2300-F* | GGAAGGTGGCTCCTACAAATGC |
| *2300-R* | CTGCTTCATGTGGTCGGGGTAG |
| *MaDFRa-F* | GAACGGTCCTCGTGGAGGAGCG |
| *MaDFRa-R* | TTCTGCCGCTCCTCCACGAGG |
| *MaDFRb-F* | CAGAAGCCGGAATATGATCTGA |
| *MaDFRb-R* | ACTCCATGCGCTCAGATCATATTCC |
| *MaDFRc-F* | GAACGGTCCTCGTGGAGGAGCG |
| *MaDFRc-R* | ACTCCATGCGCTCAGATCATATTCC |
