# Supplementary material for: Cloning and Functional Characterization of Dihydroflavonol 4-Reductase Gene Involved in Anthocyanidin Biosynthesis of Grape Hyacinth
Source: Int J Mol Sci. 2019 Sep 24;20(19):4743. doi: 10.3390/ijms20194743 (PMC6801978; doi:10.3390/ijms20194743)
Supplement: Supplementary file 1 [file ijms-20-04743-s001.zip › supplementary/Supplementary Table S2.docx]

Supplementary Table S2 correlation of Pg, Cy and Dp and anthocyanin biosynthetic gene expression levels

| Anthocyanin  Gene name | Dp | Pg | Cy |
| --- | --- | --- | --- |
| CHS  CHI  F3H  F3`H  F3`5`H  **DFR**  ANS  UFGT | \| 0.399 \| \| --- \| \| -0.421 \| \| 0.812 \| \| 0.816 \| \| 0.415 \| \| **0.850** \| \| 0.649 \| \| 0.730 \| | 0.963  -0.366  0.348  0.942  0.975  0.234  0.106  0.273 | 0.652  -0.518  0.690  0.905  0.645  0.632  0.386  0.547 |
