# Supplementary figures and images for: Cloning and Functional Characterization of Dihydroflavonol 4-Reductase Gene Involved in Anthocyanidin Biosynthesis of Grape Hyacinth
Source: Int J Mol Sci. 2019 Sep 24;20(19):4743. doi: 10.3390/ijms20194743 (PMC6801978; doi:10.3390/ijms20194743)

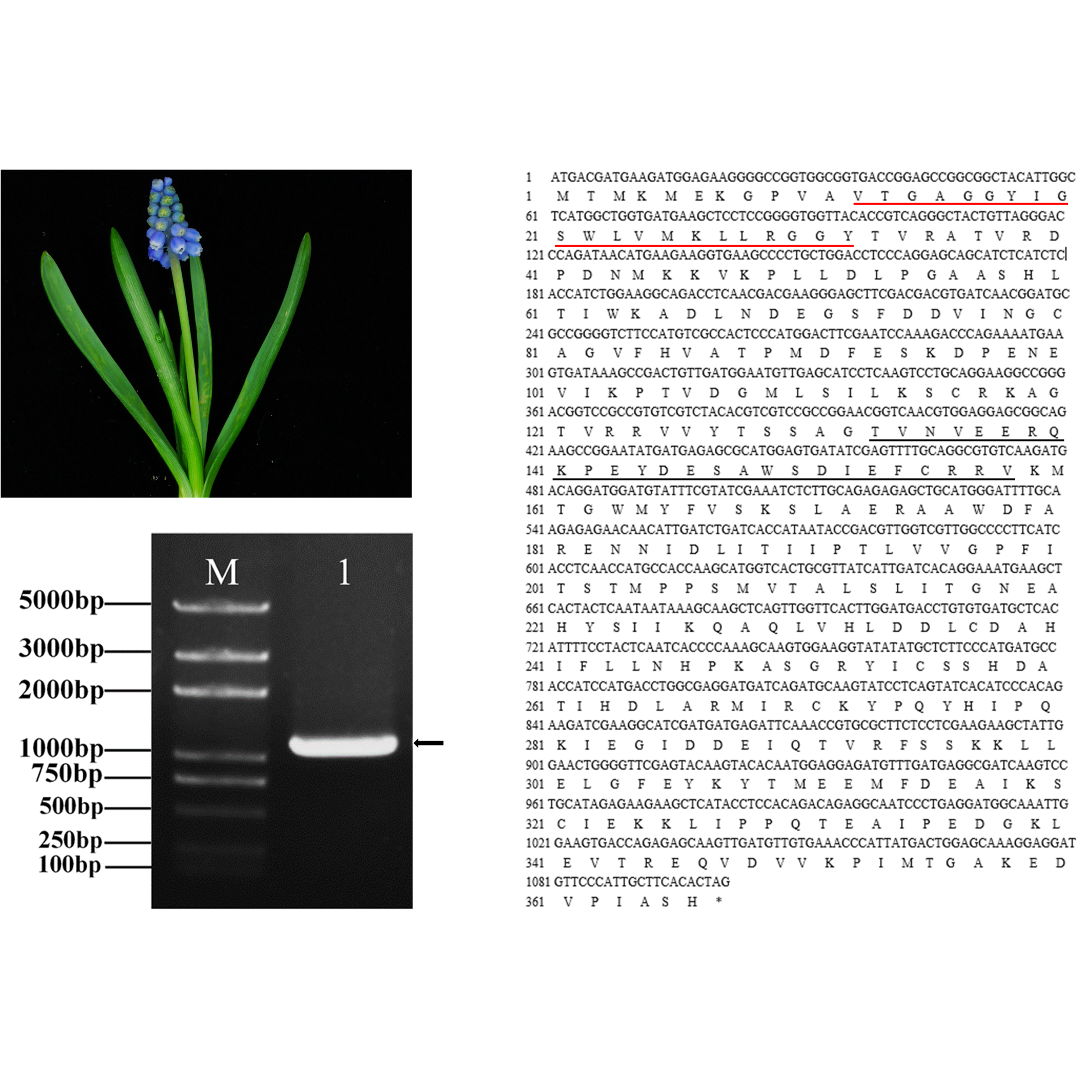

Supplement: Supplementary file 1 [file ijms-20-04743-s001.zip › supplementary/Supplementary figure S1.TIF]

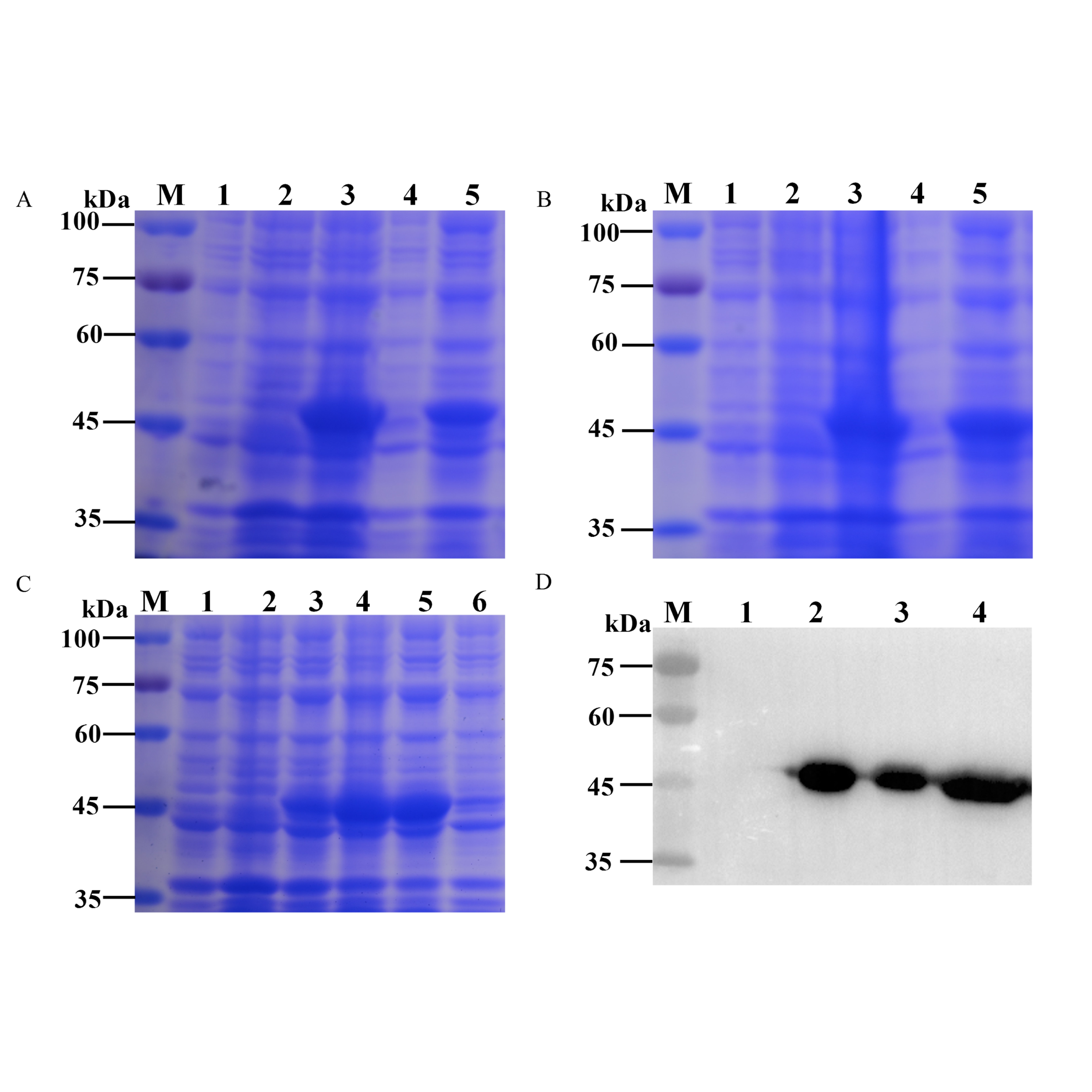

Supplement: Supplementary file 1 [file ijms-20-04743-s001.zip › supplementary/Supplementary figure S2.tif]
